# Human Replay Spontaneously Reorganizes Experience

**DOI:** 10.1016/j.cell.2019.06.012

**Published:** 2019-07-25

**Authors:** Yunzhe Liu, Raymond J. Dolan, Zeb Kurth-Nelson, Timothy E.J. Behrens

**Affiliations:** 1 Wellcome Trust Centre for Neuroimaging, University College London, London WC1N 3AR, UK; 2 Max Planck University College London Centre for Computational Psychiatry and Ageing Research, University College London, London WC1B 5EH, UK; 3 DeepMind, London, UK; 4 Wellcome Centre for Integrative Neuroimaging, Centre for Functional Magnetic Resonance Imaging of the Brain, University of Oxford, Oxford OX3 9DU, UK

**Keywords:** replay, preplay, generalization, inference, factorized representation, transfer learning, hippocampus, place cells, grid cells, MEG

## Abstract

Knowledge abstracted from previous experiences can be transferred to aid new learning. Here, we asked whether such abstract knowledge immediately guides the replay of new experiences. We first trained participants on a rule defining an ordering of objects and then presented a novel set of objects in a scrambled order. Across two studies, we observed that representations of these novel objects were reactivated during a subsequent rest. As in rodents, human “replay” events occurred in sequences accelerated in time, compared to actual experience, and reversed their direction after a reward. Notably, replay did not simply recapitulate visual experience, but followed instead a sequence implied by learned abstract knowledge. Furthermore, each replay contained more than sensory representations of the relevant objects. A sensory code of object representations was preceded 50 ms by a code factorized into sequence position and sequence identity. We argue that this factorized representation facilitates the generalization of a previously learned structure to new objects.

## Highlights

• As in rodents, human replay occurs during rest and reverses direction after reward
• As in rodents, human replay coincides with hippocampal sharp-wave ripples
• Human replay spontaneously reorganizes experience based on learned structure
• Human replay is factorized, allowing fast structural generalization

## Graphical Abstract

**Figure:**
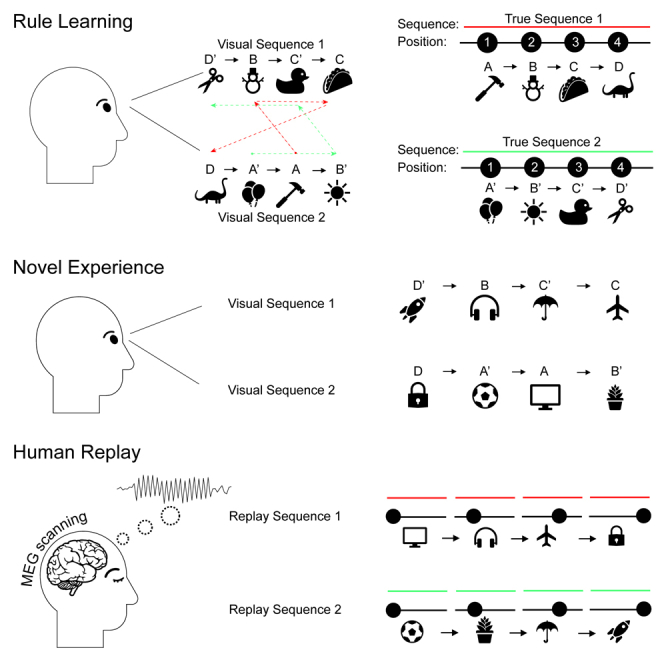

## Teaser

In the human hippocampus, neural activity known as sequential replay includes both item-specific and generalizable abstract representations and spontaneously reorders sequences to integrate past knowledge with current experiences during rest.

## Introduction

Although artificial intelligence (AI) is making impressive strides, humans still learn orders of magnitude faster (Tsividis et al., 2017). Humans are adept at making rich inferences from little data by generalizing, from past experiences, knowledge of how things relate to one another. A crashed car by the roadside, for example, conjures a detailed sequence of likely past events that were never actually witnessed. It has been theorized that such inferences rely on internal models of the world, which are supported by the same neural mechanisms underpinning relational reasoning in space (Behrens et al., 2018, Buzsáki and Moser, 2013, Constantinescu et al., 2016, Eichenbaum, 2017, Tolman, 1948).In spatial tasks, cells in the rodent hippocampal formation, including place and grid cells, encode a map of the environment (Moser et al., 2008). Although predominantly encoding one’s current location, these cells also spontaneously recapitulate old (Diba and Buzsáki, 2007, Foster and Wilson, 2006, Louie and Wilson, 2001, Skaggs and McNaughton, 1996) and explore new (Gupta et al., 2010, Ólafsdóttir et al., 2015) spatial trajectories—a phenomenon known as “replay.”

The capacity to play out trajectories (Gupta et al., 2010), and even locations (Ólafsdóttir et al., 2015), that have never been experienced suggests replay might be important for building and sampling internal models of space (Foster, 2017, Pezzulo et al., 2014). If similar mechanisms do indeed apply in non-spatial scenarios, it would provide one substrate for the powerful inferences and generalization that characterize cognition in humans, whose non-spatial reasoning capacities dwarf those of rodents. It is, therefore, intriguing that spontaneous trajectories of non-spatial-state representations can be measured in humans, time compressed to a speed similar to rodent replay (Kurth-Nelson et al., 2016).

How might replay facilitate inference and generalization? During hippocampal spatial replay events, coherent replays of the same trajectories have been recorded in both medial entorhinal cortices (mECs) (Ólafsdóttir et al., 2016) and visual cortices (Ji and Wilson, 2007). These anatomically distinct regions differ markedly in the nature of their representations. While visual representations encode the sensory properties of a particular object or event, mEC representations encode knowledge of relationships (such as spatial relationships) invariant over the things participating in the relationship. We refer to this as “structural” knowledge.

In space, strong constraints are provided by a requirement for neighborhood relationships to lie in two physical dimensions, but this is just one example of a general class of probabilistic constraints applied to the relationships between objects (Behrens et al., 2018, Tenenbaum et al., 2011). Remapping experiments suggests that entorhinal cells encode structural knowledge explicitly—divorced from sensory representations—thereby enabling the same relational constraints to be applied to different sensory environments (Fyhn et al., 2007, Whittington et al., 2018, Yoon et al., 2013).

Keeping structural knowledge independent of particular sensory objects is a form of factorization. Factorization (also called “disentangling”; Bengio et al., 2013, Higgins et al., 2017a) means that different coding units (e.g., neurons) represent different factors of variation in the world. This contrasts with a non-factorized representation in which units code for mixtures of variables (e.g., conjunctive codes in hippocampus; Komorowski et al., 2009). Factoring abstract structures from particular instances can, in principle, enable old structural knowledge to impact seamlessly on new learning (Behrens et al., 2018, Friston and Buzsáki, 2016). One intriguing possibility is that factorization also enables immediate constraints on the replay of new sensory experiences, providing a potential mechanism for novel inferences. Approaching drivers, for example, may see the crashed car first, then the road ice, and then the arriving ambulance, but nevertheless replay events in the correct order.

In this paper, we first investigate the relationship between human non-spatial sequences and rodent hippocampal replay. We then hypothesize that if replay is important for rapid inference, it should re-order events according to previously learned structural information, rather than merely recapitulating the sequence of experience. To support this inference, we hypothesize that replayed representations are factorized into structural information (common across related experiences) and sensory information (unique to each experience).

**Figure S1:**
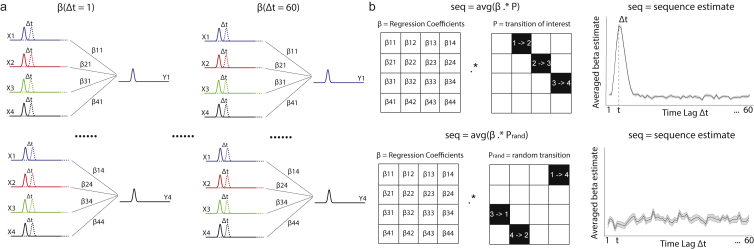
Illustration of Sequenceness Analysis, Related to Figure 1 a, The predictor matrix, X, for this regression is the same matrix, Y, but *time-lagged by Δt* (see inset) to search for linear dependencies between state activations at this time-lag. We constructed separate prediction matrices for each Δt. We then performed time-lagged regression by regressing each lagged predictor matrix X(Δt) onto the state reactivation matrix, Y, resulting in a regression coefficient matrix, with dimension of *s* states ^∗^*s* states, at each time lag. b, This coefficient matrix was then projected onto the hypothesized state transition matrix P, to give a single measure of sequenceness as a function of time-lag (Δt), and transition structure (P). Evidence of sequenceness for transition of interest (ground truth) versus random transitions were shown on the top and lower panel respectively. Notably, this regression approach allowed us to include confound regressors in the analysis. We found it helpful to include lagged time-courses at Δt+100ms, Δt+200ms … as confounds to account for 10Hz oscillations that are prevalent during resting activity.

To test these hypotheses, we conducted two studies in human participants, using magnetoencephalography (MEG) to measure fast spontaneous sequences of representations (described in the Method Details and Figure S1). In each study, we first trained participants on a rule that defined a permutation over a sequence of objects. We then presented a novel set of stimuli, but in an order that differed from the order implied by the structure. During a subsequent period of rest, we found rapid replay of trajectories following the rule defined as opposed to the experienced order. As in rodents (Ambrose et al., 2016), a preponderance of reverse trajectories along a sequence greatly increased after that sequence was rewarded. Within a replay event, representations of objects were preceded by abstract factorized codes reflecting both the sequence identity and the object position within a sequence. Finally, analogous to preplay, spontaneous sequences of an abstract structure representation played out even before the actual experience with the objects.

## Results

### Study 1: Unscrambling New Objects Using a Previously Learned Rule

**Figure 1:**
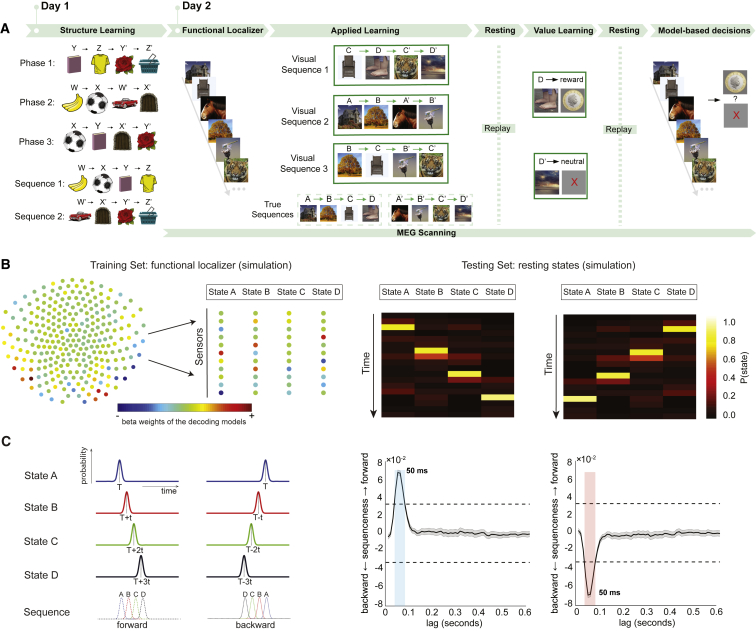
Task Design of Study 1 and Sequenceness Measurement (A) Participants were presented with visual stimuli where the correct sequences were scrambled (study 1). Subjects were pre-trained on day 1 to re-assemble the stimuli into a correct order. On day 2, participants underwent a MEG scan while performing a task with the same structure but different stimuli. (B) Using functional localizer data, a separate decoding model (consisting of a set of weights over sensors) was trained to recognize each stimulus (left). Decoding models were then tested on unlabeled resting data. Examples of forward and reverse sequential stimulus reactivations are shown in simulated data (right). (C) “Sequenceness,” based on cross correlation, quantifies the extent to which the representations decoded from MEG systematically follow a transition matrix of interest (left). Evidence for sequenceness (y axis) was quantified at each time lag independently (right) for all possible time lags up to 600 ms (x axis). The dashed line indicates a nonparametric statistical significance threshold (see Method Details). The gray area indicates the standard error across simulated participants. Colored areas mark the lags at which the evidence of sequenceness exceeded the permutation threshold in the forward (blue) or reverse (red) direction. All data in this figure are from a simulation where sequences were generated with a state-to-state lag of 50 ms. See also Figures S1, S2, and S3.

We first tested whether replay-like activity is informed by abstract knowledge generalized from previous experiences. This necessitated a task design wherein learned sequential structure can be applied to novel sensory stimuli to infer a new ordering. To accomplish this, we designed a novel behavioral task, with links to both sequence learning and sensory preconditioning (Figure 1) (Dehaene et al., 2015, Wimmer and Shohamy, 2012).

On day 1, participants viewed eight pictures. The pictures appeared sequentially, but participants were aware that this order of appearance was a scrambled version of a different sequential order (the “true” order), which would be crucial for obtaining a reward later. The underlying true order contained two sequences. The scrambled sequence presented to subjects consisted of three stages, and each stage contained one transition from each true sequence. For example, the true sequences WXYZ and W’X’Y’Z’ might be presented in these three stages: [YZ, Y’Z’], [XY, X’Y’], and [WX, W’X’]. The interval within a pair was 300 ms, and the interval between pairs was 900 ms. This scrambling rule was consistent for each subject. Before viewing the scrambled sequences, participants were carefully instructed on the rule that unscrambled the six visual transitions into the two true sequences. Here, the rule is that seeing YZ, XY, and WX implies a true sequence is WXYZ. Further details are found in the Method Details.

**Figure S2:**
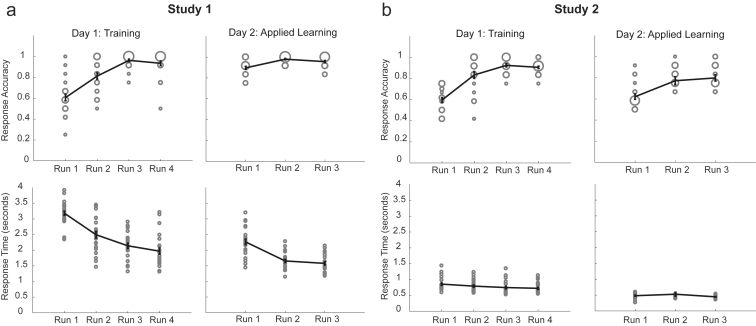
Behavioral Performance during Training on Day 1 and Applied Learning on Day 2, Related to Figures 1 and 4 After learning the structure, participants could quickly unscramble the sequences of different images in both studies. a, In Study 1, during training on Day 1, the response accuracy on the sequenceness quiz increased gradually over runs, and the responses time decreased over runs, while on Day 2, during applied learning of different stimuli, most of the participants understood the correct sequence immediately after first run, and responses were already fast in the first run. b, In Study 2, similar effects were found. Note there was a stricter time limit on responses in Study 2 (2 s during training on Day 1, and 600 ms during applied learning on Day 2) compared to Study 1 (5 s during both Day 1 training, and Day 2 learning), which makes the absolute accuracy values not directly comparable. Each circle indicates one unique value. The size of the circle corresponds to the number of participants who have the same value.

On day 2, during MEG scanning, we presented eight new pictures comprising six transitions—[CD, C’D’], [AB, A’B’], and [BC, B’C’]—in a scrambled order that adhered to the same rule learned on day 1 and with the same timings. We refer to this phase as “applied learning.” Participants were quizzed on the true order after each stage, without feedback. Accuracy at this task stage was 94.44% (vs chance 50%, p < 0.0001; Figure S2A), indicating a correct application of the previously learned rule to these new objects. After this, participants were shown that the terminal object of one sequence, either D or D’, was associated with a reward, thereby establishing one sequence as rewarding and one as neutral. We call this phase “value learning.” Participants were then shown random objects from the sequences and asked whether that object was part of a sequence that would lead to the reward. No feedback was provided during these questions, to preclude further learning. Overall accuracy in this phase was 98.55%, indicating a correct application of the learned transition model.

### Neural Activity Spontaneously Plays Out Sequences of New Stimuli in an Inferred Order

Between the applied learning and value learning phases, there was a resting period of 5 min with no task demands or visual inputs. In this resting period, we looked for spontaneous neural sequences following either the order of visual experience or an order defined by the previously learned structure. The rest period was intended to be analogous to the awake resting periods in rodent studies in which hippocampal replay has been observed in spatial tasks (Davidson et al., 2009, Foster and Wilson, 2006, Karlsson and Frank, 2009).

To index replay in spontaneous brain activity during rest, we needed to be able to decode visual stimuli from patterns of MEG sensor activity (analogous to decoding the location from hippocampal cellular activity). Therefore, we included a functional localizer task before the main task. Placing the functional localizer before the main task ensured there was no task-related information associated with these training data. Here, participants simply saw the images that would later be used in the task, presented in a random order, and prior to the acquisition of knowledge regarding which visual object played which role in a sequence.

**Figure S3:**
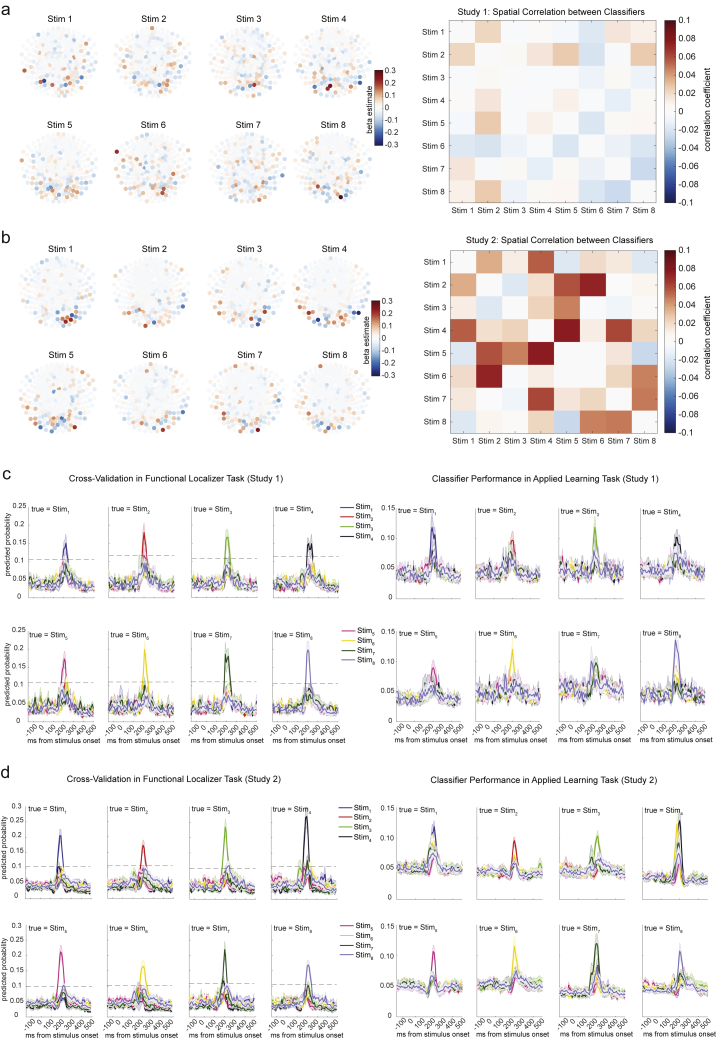
Sensor Maps, Spatial Correlation, and Classifiers Performance of Trained Lasso Logistic Regression Models, Related to Figures 1 and 4 a, Sensor map for each state decoding model in Study 1 is shown on the left, with correlation matrix between classifiers shown on the right. b, Sensor maps and correlation matrix is shown for Study 2. c, In study 1, leave-one-out cross-validation results for each classifier in functional localizer task is shown on the left. Dotted line indicates the permutation threshold estimated by randomly shuffling the labels and re-doing the decoding process. Classifier performance during applied learning is shown on the right. These plots only use classifiers trained at 200 ms post stimulus onset. The x axis refers to the time point used for testing the classifiers. The curves therefore have a different shape than plots made by varying both the training and testing time. d, Study 2 had a very similar pattern.

We trained lasso logistic regression classifiers to recognize the patterns of brain activity evoked by each visual object. We trained one binomial classifier to recognize each object. Models were cross validated on training data through a leave-one-out approach to confirm they captured essential object-related features in the MEG signal. Peak decoding accuracy was at 200 ms post-stimulus onset (37.16% ± 3.0%), and hereafter for all analyses, we used the classifiers trained at 200 ms post-stimulus onset. We found no significant spatial correlation between trained classifiers (highest correlation *r* < 0.1; Figure S3A). The probabilities predicted by each trained model on held-out data exceeded a significance threshold only when the true stimulus was the same as what that model was trained to detect (Figure S3C; classifier performances on the applied learning task were also shown on right).

We then applied these trained classifiers to the resting period following the applied learning phase. This produced a reactivation probability of each object at each time point during rest (Figure 1B). Next, we quantified the degree to which these reactivation probabilities systematically followed particular sequences (Figure 1C) using a measure of “sequenceness” (described in detail in the Method Details and Figure S1). This measure defines the extent to which reactivations of object representations follow a consistent sequence defined by a transition matrix.

We considered two transition matrices: the order of visual experience (e.g., C → D → C’ → D’) and the true sequences defined by the underlying rule (e.g., A → B → C → D). Significance was tested by randomly shuffling the transition matrix of interest many times and repeating the same analysis to generate a null distribution of sequenceness. We took the peak of the absolute value of each shuffle across all lags as our test statistic to correct for the multiple comparisons at multiple lags. For a significance threshold, we used the (absolute) maximum of these peaks across time (dashed line in Figure 1B).

**Figure 2:**
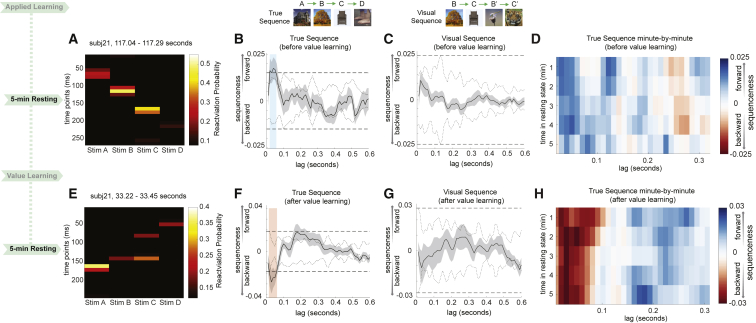
Replay Follows A Rule-Defined Sequence and Reverses Direction after Value Learning (A and E) In study 1, examples of sequence events during rests before (A) and after (E) value learning are shown from one subject for visualization purposes. Each row depicts reactivation probabilities at a given time point. For statistical purposes, data were analyzed using a “sequenceness” measure (see Method Details for details). (B and C) Stimulus representations decoded from MEG spontaneously played out sequences following a structure defined by the previously learned rule (B) and not the visually experienced sequence (C). The dotted line is the peak of the absolute value of all shuffled transitions, separately at each time lag; the dashed line is the max across all time lags, which we use as a corrected threshold. During the first rest period, the rule-defined sequences played in a forward direction. (D) Forward replay of the rule-defined sequence appeared in the first min of the resting period and remained stable for 4 min. (E) In the second rest period, the rule-defined sequences reversed direction to play in a backward order. Shown is an example sequence event. (F and G) As in the first rest period, there was statistical evidence for replay of the rule-defined sequence (F) but not the order of the visual experience (G). (H) Reverse replay of the rule-defined sequence after value learning was stable for all 5 min of rest. Blue indicates forward sequenceness, and red indicates reverse. See also Figure S4 and S6.

**Figure S4:**
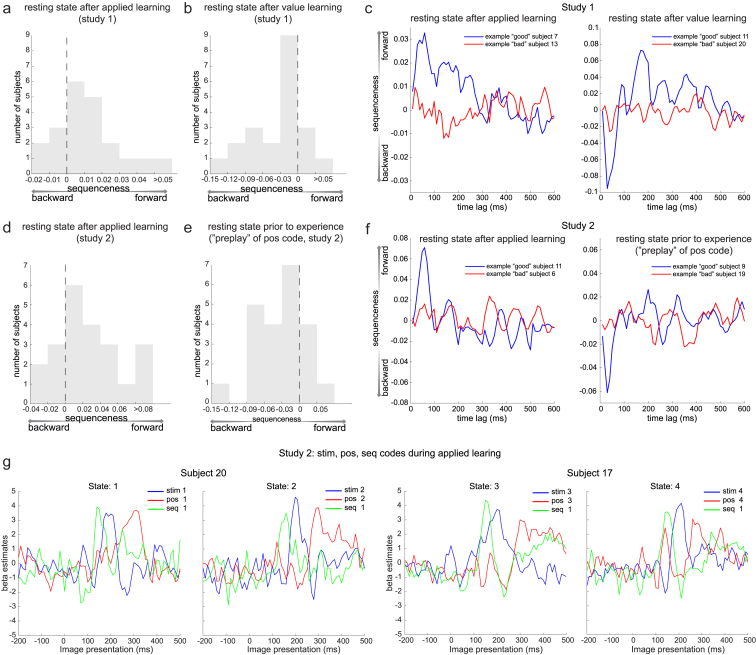
Sequenceness Distribution across Subjects and Example Data, Related to Figures 2, 4, 5, and 6 a, From Study 1, during rest after applied learning but before value learning, 16/21 subjects had forward sequenceness at 40 ms time lag. b, Following value learning in Study 1, 17/21 subjects showed reverse sequenceness. c, Sequenceness plot from examples of one “good” subject and “bad” subject in Study 1 are shown both for resting before (left) and after (right) value learning. d, From Study 2, forward replay of the true sequence after applied learning was evident in 17/22 subjects. e, The position code was played (in reverse direction) prior to experience with the stimuli in 17/22 subjects. f, Sequence plot from examples of one “good” subject and “bad” subject in Study 2 are shown both for preplay and replay. g, Examples of three codes: *stim*, *pos*, and *seq* codes reactivation from two subjects during applied learning in Study 2 were shown for visualization purpose. These plots (and similar ones for the whole group in Figure 5A) show results of a multiple linear regression of the 3 sensor maps associated with the current image (blue), the current position (red) and the current sequence (green) onto the sensor time courses measured after an image is presented during learning. The sensor time courses first represent the sequence, then the image, then the position in the sequence.

Figure 2A shows example sequential stimulus reactivations during the rest period following applied learning. We found evidence of sequential neural activity that conformed to the rule-defined, (Figure 2B) but not the visually experienced, order (Figure 2C). These sequences were predominantly in a forward direction. The effect exceeded the permutation threshold from 20 to 60 ms of stimulus-to-stimulus lag (p < 1/24 ≈ 0.042, corrected), peaking at 40 ms (n = 21, resting after applied learning, β = 0.017 ± 0.005, mean ± SEM). Sequenceness appeared within the first min of rest (Wilcoxon signed-rank test, p = 0.016), remained stable over the next 4 min (second min, p = 0.004; third min, p = 0.332; fourth min, p = 0.027; fifth min, p = 0.709; Figure 2D) and was present in the majority of participants (Figure S4A; examples of individual sequences in Figure S4C, left).

### Direction of Spontaneous Sequences Reverses after Reward

In rodents, rewards increase the relative frequency of reverse, but not forward, replays (Ambrose et al., 2016). Following the value learning phase, participants had another 5-min resting period. This second resting period allowed us to test for reward-induced increases in reverse sequences in humans. Figure 2A shows example sequential stimulus reactivations during the second rest period following value learning. Applying the same sequence analysis as above, we found the direction of spontaneous neural sequences switched from forward to reverse (Figure 2F), exceeding the permutation threshold from 20 to 70 ms of stimulus-to-stimulus lag, and again peaking at 40 ms (n = 21, resting after value learning, β = −0.028 ± 0.010). Again, there was no evidence for sequences along visual experienced trajectories (Figure 2G). The reverse sequenceness effect appeared within the first min of rest (Wilcoxon signed-rank test, p = 0.045), persisted for all 5 min (second min, p = 0.003; third min, p = 0.192; fourth min, p = 0.0047; fifth min, p = 0.159; Figure 2H), and was seen for most participants (Figure S4B; examples of individual sequences in Figure S4C, right).

**Figure 3:**
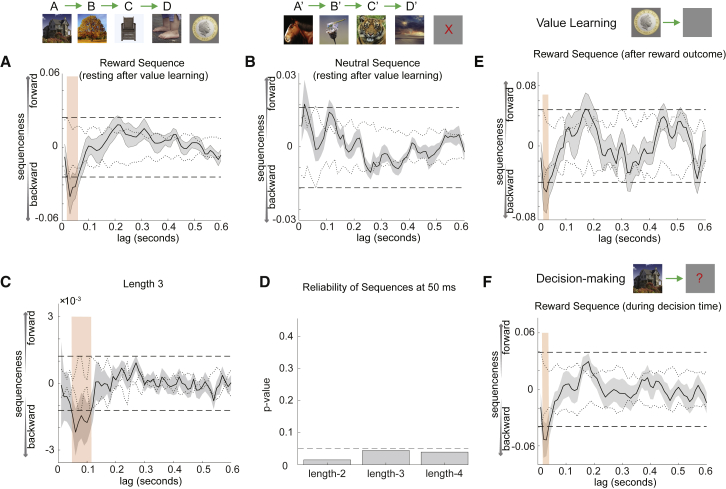
Only Rewarded Sequences Reverse Direction, and Sequences Form Chains of Four Objects (A) In value learning, each participant experienced one rewarded sequence and one unrewarded sequence. In the rest period after value learning, the rewarded sequence played backward in spontaneous brain activity. (B) The unrewarded sequence still trended to playing forward. (C and D) All other panels in the main text show a sequenceness measure that evaluates single-step state-to-state transitions. Here, we report a related sequenceness measure that evaluates the *extra* evidence for multi-step sequences, beyond the evidence for single-step transitions (see Method Details for details). This measure describes the degree to which, for example, A follows B with the same latency as B follows C. Sequences of length 3, following the rule-defined order, played out at a state-to-state lag of approximately 50 ms (C). At 50-ms lag, there was significant replay of sequences up to the maximum possible length (D → C → B → A). Dashed line at p = 0.05 (D). (E) Replay was not limited to the resting period. Reverse replay of the rewarded sequence began during value learning. (F) Reverse replay of the rewarded sequence was also evident during the decision phase.

When we examined rewarded and neutral sequences separately, we found evidence that the rewarded sequence alone reversed direction (Figure 3A), peaking at 40 ms (β = −0.052 ± 0.018), and the neutral sequence remained dominantly forward (Figure 3B), peaking at 20 ms (β = 0.021 ± 0.011). Interestingly, reverse replay of rewarding sequences was already present in the value learning phase immediately after seeing the rewarding outcome (i.e., money coin, peaking again at 40 ms, β = −0.053 ± 0.016; Figure 3E). It also continued during the subsequent decision task (peak at 40 ms, β = −0.055 ± 0.020 for reward trials; Figure 3F).

### Length-n Sequences

Although the transition matrix defined by the order of visual presentation (e.g., C → D → C’ → D’) differed from the transition matrix defined by the rule (e.g., A → B → C → D), some individual pairwise transitions were common between the two (e.g., C → D). Therefore, we would expect our sequenceness measure to detect some “rule-defined sequenceness” even if the brain replayed the order of visual presentation alone. The fact that there was greater evidence for the rule-defined sequence than the visually observed sequence renders this interpretation unlikely. However, to directly rule out this possibility we sought evidence for length-3 or length-4 sequences. We defined a measure of length-n sequenceness that controlled for all lengths up to n-1, measuring the additional evidence for sequences of length-n (see Method Details for details).

In the task, there was no overlap between rule-defined sequences (e.g., B → C → D) and visual-order sequences at length-3, meaning length-3 rule-defined sequences could only be reliably observed if neural sequences truly followed a rule-defined ordering. Indeed, we found significant evidence for length-3 reverse replay of rule-defined sequences (Figure 3C), peaking at 50-ms stimulus-to-stimulus lag (n = 21, resting after value learning, β = −0.0022 ± 0.0010). The additional evidence for length-4 rule-defined sequences was also significant at 50-ms time lag (β = −0.00023 ± 0.00011; Figure 3D). Together, these data suggest that rapid sequences of non-spatial representations can be observed in humans and have characteristics of hippocampal replay events recorded in rodents. Furthermore, these replay events play out sequences in an order that is implied but never experienced.

### Study 2: Scrambling Individual Transitions

**Figure 4:**
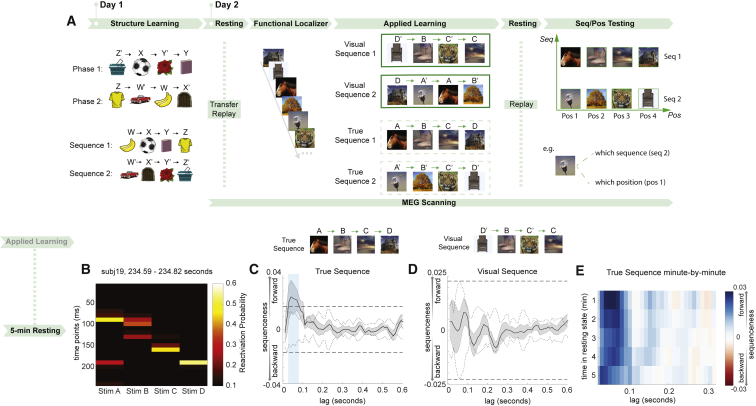
Task Design of Study 2 and Replication of Replay Following Rule-Defined Sequence (A) In study 2, not only were the sequences scrambled (as was the case in study 1), but additionally, the pairwise associations were no longer preserved. On day 1, participants were pre-trained on the rule that defined a re-ordering between the order of stimulus appearance and the task-relevant order. They were also instructed that this same mapping would pertain to novel stimuli on day 2. On day 2, participants were shown the novel stimuli while undergoing a MEG scan. (B) Example of forward-replay sequence event from one subject. (C) During the second rest period, there was statistical evidence for forward replay of the rule-defined sequence. (D) There was no evidence for replay of the visually experienced sequence, as in study 1. (E) Forward replay of the rule-defined sequence was stable for all 5 min of rest. See also Figures S2, S3, S4, S5, and S6.

In study 1, we found replay-like activity plays out events in an implied but never actually experienced order. However, in that study, participants experienced each individual transition of the implied sequence, albeit not contiguously. Thus, one possibility is that the observed ABCD sequence in brain activity could have arisen from a Hebbian-like encoding of individual transitions. In other words, if B and C were associated, and separately A and B were associated, then ABC could be played out through a serial Hebbian-like associative mechanism. To rule out this hypothesis and determine whether replay truly incorporates abstract structural knowledge transferred from previous training, we needed a more rigorous test that unambiguously distinguished this hypothesis from a simpler associative mechanism. Therefore, in study 2 we designed a task similar to that of study 1, but with a new scrambling rule where pairwise transitions themselves were disrupted, so that correct sequences could only be inferred using structural knowledge (Figure 4A).

On day 1, before training, participants were explicitly instructed on the mapping between the order of visual presentation and the sequence position of all stimuli. For example, the first presented stimulus might belong to the second position of the first sequence. This mapping allowed participants to put stimuli in the right order. After that, participants went through four runs of training, with two phases in each run. Each phase was presented three times, with an equal time gap of 500 ms between each stimulus. For example, in the first stage, participants observed pictures Z’, X, Y’, and Y, with a 500-ms fixation period prior to each stimulus. In the second stage, they observed pictures Z, W’, W, and X’. These eight objects again formed two true sequences: WXYZ and W’X’Y’Z’. The mapping between presentation order and unscrambled sequence of the eight objects was now entirely random (and different across participants). After each run, participants were quizzed about the true sequences without feedback, as in study 1. To pass the training, they were required to reach at least 80% accuracy on average (see Method Details for more details).

Day 2 took place in the MEG scanner and used the same unscrambling rule as day 1, but applied now to novel objects. Similar to study 1, participants were quizzed on the true order after each run (three runs in total) without feedback. Average accuracy was 73.1% (p < 0.0001 vs chance), indicating correct application of the previously learned rule to these new objects (Figure S2B). Note the slight drop in accuracy compared to study 1 is likely due to a much stricter time limit of 600 ms.

Unlike study 1, in study 2, participants were given a 5-min rest period before any visual exposure to the objects. Participants then performed a functional localizer task where object stimuli were presented in a randomized order. Next, these same stimuli were presented in the scrambled order described above. This was followed by another 5-min resting period. At the very end, participants were shown the stimuli again in a randomized order but were now asked to perform one of two judgments on each stimulus as it appeared. On *position judgment* trials, they were asked to indicate the position (i.e., position 1, position 2, position 3, or position 4) of the stimulus within the sequence it belonged to. On *sequence judgment* trials, they were asked to indicate which sequence (i.e., sequence 1 or sequence 2) the stimulus belonged to. Study 2 had no reward component.

### Neural Sequences Infer a New Order, even when Pairwise Transitions Are Disrupted

**Figure S5:**
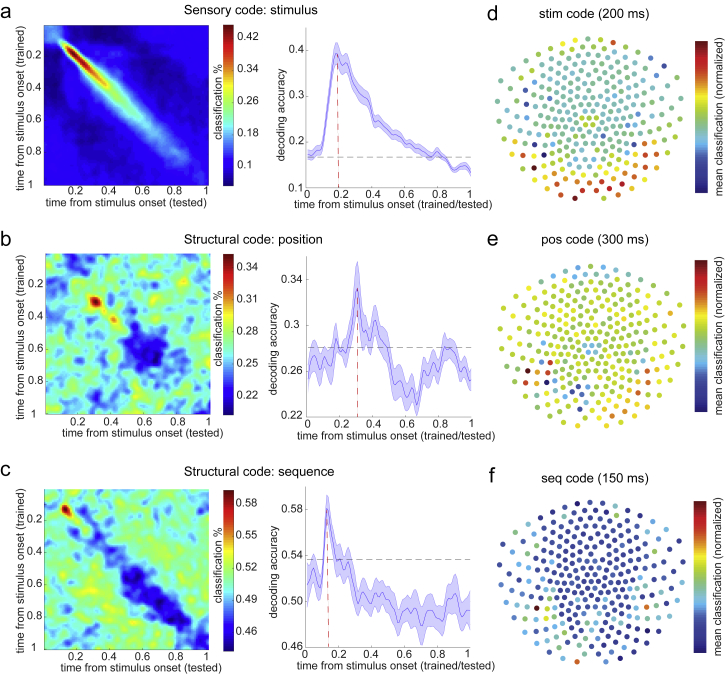
Multivariate Decoding for Sensory and Structural Representations, Related to Figures 4, 5, and 6 a, Using functional localizer data, we trained decoders for the sensory level representation with a leave-one-out cross-validation approach. There was a peak in accuracy at 200 ms post stimulus onset, consistent with previous findings. b, To find the peak time point to train position decoders, we trained classifiers on every time point relative to the onset of stimuli, and tested at every time bin relative to the same onsets in the sequence testing block. Each cell of this grid shows cross-validated prediction accuracy on average (left panel). We used the sequence rather than position testing block because of the alignment between the positions and motor responses in the task. Right panel shows the diagonal of the left panel matrix. The peak decoding time was 300 ms after stimuli onset. Dashed lines show 95% of empirical null distribution obtained by shuffling state labels. Shaded area shows standard error of the mean. c, Same procedure applied to find the peak time point for sequence identity decoders. This was done on the position testing block to avoid a motor response confound. The peak decoding time was 150 ms after stimulus onset. Structure and sensory codes have distinct encoding in the brain. Averaged decoding accuracy across subjects with each sensor (bootstrapping, n = 2000, with 50 sensors each time) is shown: *stim* decodes individual stimuli (d); *pos* decodes order within sequence, invariant to which sequence (e); *seq* decodes which sequence, invariant to which stimulus within sequence (f).

As in study 1, we trained a set of classifiers to recognize individual objects from study 2 using data from the functional localizer. We call these representations the “stimulus code.” (Note that we use the term “code” to refer to aspects of brain-derived signals that are related to quantities of interest.) The validity of these classifiers was again tested using leave-one-out cross validation. Decoding accuracy peaked at 200 ms post-stimulus onset (39.6% ± 2.2%; Figure S5A). Sensor maps, spatial correlations, and classification accuracies for the 200-ms classifier are shown in Figures S3B and S3D.

**Figure S6:**
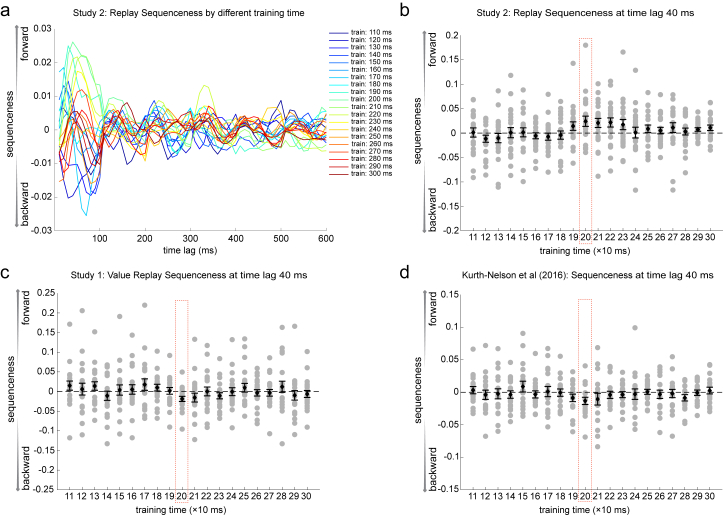
Sequenceness Analysis with Classifiers Trained at Different Times, Related to Figures 2 and 4 a, Sequenceness using classifiers trained at different times relative to stimulus onset (110 ms – 300 ms). This is from resting data after applied learning in Study 2. b, Scatterplot of the sequenceness at 40 ms time lag as a function of classifier training times in Study 2 during rest period after applied learning. 200 ms is the training time used throughout the current study. c, Scatterplot of the sequenceness at 40 ms time lag as a function of classifier training time in Study 1 during rest period after value learning. d, A similar result was obtained by re-analyzing data from Kurth-Nelson et al. (2016). Notably, there is a preference for replay of a particular time-slice of the representation (at 200 ms) in all 3 studies. That is, although the representation of each stimulus evolves over a period of approximately 500 ms, only one time-slice (at 200 ms) reliably replays during rest (with current data analysis approaches). This time-slice (200 ms) is consistent across 3 independent datasets.

Using these stimulus code classifiers (trained at 200 ms post-stimulus, as in study 1, but see Figure S6 for other training times), we first examined data from the resting period following the applied learning phase. Examples of sequential stimulus reactivations are shown in Figure 4B. As in study 1, we found evidence for forward sequenceness following a rule-defined transition matrix (Figure 4c), but not the transition matrix of visual experience (Figure 4D). This effect again peaked at a stimulus-to-stimulus lag of 40 ms (exceeding the permutation threshold from 30 to 70 ms, n = 22, β = 0.021 ± 0.009); appeared within the first min of rest (Wilcoxon signed-rank test, p = 0.0002); remained stable for all 5 min (second min, p = 0.007; third min, p = 0.001; fourth min, p = 0.006; fifth min, p = 0.016; Figure 4E); and existed in the majority of participants (Figure S4D; examples of individual participants in Figure S4F, left). Unlike study 1, however, this stimulus order could not have emerged from simple associative mechanisms, as no correct pairwise associations were present in the visually experienced sequence (Figure 4A; cf. Figure 1A). Therefore, we argue that this reordering implies a transfer of structural knowledge from the previous day.

We found no correlation in either study between replay strength and behavioral performance in the learning task (*r* = −0.16, p = 0.48 in study 1; *r* = 0.12, p = 0.60 in study 2). We speculate that this lack of correlation could be due to a ceiling effect. At the end of the experiment, we also asked participants to write down the two unscrambled sequences. All participants were 100% accurate, suggesting they had a perfect knowledge of the mapping.

### Neural Representations Embed Structural Knowledge in a Factorized Code

The impact of structural knowledge on replay order raises a question as to whether structural knowledge itself might be a component of the replayed representation. In rodents, entorhinal grid cells replay coherently with hippocampal place cells (Ólafsdóttir et al., 2016, Ólafsdóttir et al., 2017; although see O’Neill et al., 2017). Unlike place cells, however, grid cells encode position explicitly, with a representation that generalizes across different sensory environments (Moser et al., 2008). In our task, objects do not have positions in space, but do have positions within an inferred sequence. Analogously, these positions might be represented explicitly during replay. For example, the second items of each inferred sequence would share part of their replayed representations not shared by the third items. Similarly, we wondered whether items belonging to a particular sequence (e.g., sequence 1) would share representations absent in the other sequence (e.g., sequence 2).

Therefore, in addition to stimulus code classifiers, we trained two sets of logistic regression classifiers. One was trained to recognize the position of each stimulus within its respective sequence, regardless of which sequence it belonged to. We call this the “position code.” The other was trained to recognize which sequence each stimulus belonged to, regardless of which position it occupied within that sequence. We call this the “sequence code.” Using a cross-validation approach that ensured the code was free from sensory and motor confounds (see Method Details), decoding accuracy peaked at 300 ms for the position code (33.25% ± 2.3%) and 150 ms for the sequence code (58.11% ± 1.2%; Figures S5B and S5C; see Method Details for details). As an extra precaution against the contamination of position and sequence codes by coincidental common sensory features, we regressed out the corresponding stimulus code from each position and sequence classifier. We observed that the structural and sensory codes were encoded with a different topography in the brain, with the stimulus code most strongly represented in occipital sensors (Figure S5D), while position and sequence codes were reflected more strongly in posterior temporal sensors (Figures S5E and S5F; see Method Details for details; cf. Hsieh et al., 2014).

**Figure 5:**
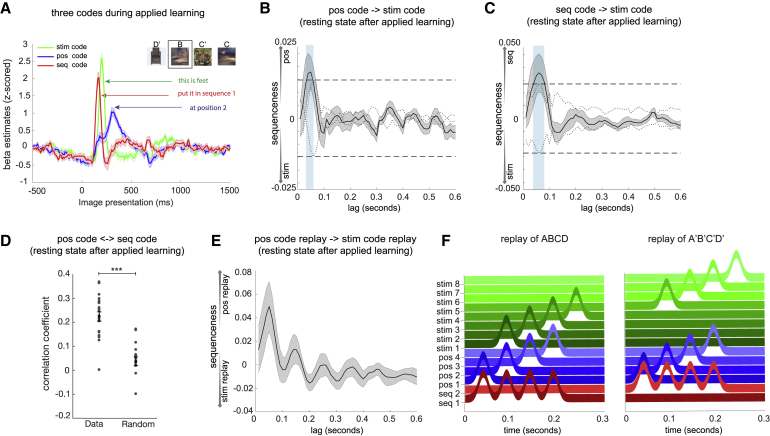
Abstract Factorized Codes Play Out in Synchrony with Replay In addition to the decoding models trained to detect representations of individual objects (*stim* code), we also trained models to detect representations of “position within sequence” (*pos* code) and “which sequence” (*seq* code). See Method Details for details. (A) During the applied learning phase of study 2, representations of *seq*, *stim*, and *pos* codes for the presented stimulus peaked at distinct times relative to stimulus onset. (B and C) During the second resting period of study 2, spontaneous reactivation of both *pos* and *seq* codes consistently preceded spontaneous reactivation of the corresponding *stim* code, with ∼50-ms lag. (D) As a validation, we also directly measured the relative timing of *pos* and *seq* activation and found a peak at 0 ms of lag; ^∗∗∗^p < 0.001. (E) Finally, we examined the temporal relationship between replay of *stim* codes (as shown in Figure 4B) and replay of *pos* codes (see Method Details for details). We found that *pos* code replay preceded *stim* code replay events by 50 ms. (F) Summary of results. During each replay event, stimulus representations (green) were preceded 50 ms by abstract sequence (red) and position (blue) representations. See also Figures S4 and S5.

Next, we asked how representations of structure and sensory information were related. We first used the three sets of classifiers (stimulus code, position code, and sequence code) to probe neural representations during the applied learning phase. We found significant activation of all three codes at times closely aligned to their respective training data: the sequence code at 150 ms (β = 2.00 ± 0.12, p < 0.0001), the stimulus code at 200 ms (β = 2.59 ± 0.1, p < 0.0001), and the position code at 300 ms (β = 1.02 ± 0.09, p < 0.0001) after stimulus onset (Figure 5A; examples from individual subjects in Figure S4G). Hence, when a new stimulus appears, neural activity encodes the unique identity of the object, its sequence position, and its sequence identity in a factorized representation. This type of representation implies that the same position code is used in different sequences with different objects, providing a potential mechanism for generalization of structural knowledge to support novel inferences.

### Abstract Representations of Sequence Identity and Position Consistently Precede Object Representations during Replay Events

Are structural representations spontaneously reactivated at rest? To address this, we applied the trained position and sequence code classifiers to the MEG data from the second resting period. As with the stimulus code classifiers, each classifier produced a time series of predicted probabilities. We first examined the temporal relationships among activations of the stimulus, position, and sequence codes. Using the sequenceness analysis described previously, we found that both the position and sequence codes were systematically activated 40−60 ms before the corresponding stimulus code (Figures 5B and 5C). This implies that the position and sequence codes were co-activated during rest. Indeed, the zero-lag correlations between the unshuffled position and sequence codes were significantly higher than the shuffled correlations (Figure 5D; two-tail paired t test, *t* (20) = 6.47, p < 0.0001).

These results show that structural representations consistently precede their corresponding object representations during rest. This raises the possibility that the reactivation of a position code could lead an object representation to replay at the correct position within a sequence. To test this idea, we first estimated the replay onset of position codes (e.g., position 1 → position 2) by multiplying the decoded probability of the first position (e.g., position 1) by the time-shifted probability of the second position (e.g., position 2; see Method Details for details). We similarly obtained the time course of the corresponding stimulus code replay. After that, we performed sequenceness analysis on the estimated time courses of position code replay and stimulus code replay, asking whether the replay of position codes has a temporal relationship to the replay of corresponding stimulus codes. We found that the replay of position code temporally led the replay of stimulus code, with a peak at 50 ms of lag (non-parametric one-sample Wilcoxon sign rank test against zero, p = 0.013; Figure 5E). These results are consistent with a model outlined in Figure 5F, where each individual object representation in a replay event is preceded by both sequence and position representations. We speculate that these abstract structural representations contribute to retrieving the correct object for the current place in a sequence.

### Abstract Position Representations Play in Sequences Prior to New Object Experience (“Transfer Replay”)

In rodents, prior to experience with a novel environment, hippocampal place cells play out spontaneous trajectories, which later map onto real spatial trajectories when the environment is experienced (Dragoi and Tonegawa, 2011). This has been called “preplay” and is proposed to encode general information about the structure of space. Our task allowed us to ask a similar question: whether abstract representations of task structure, defined using day 2 objects, are played before those objects are ever seen.

**Figure 6:**
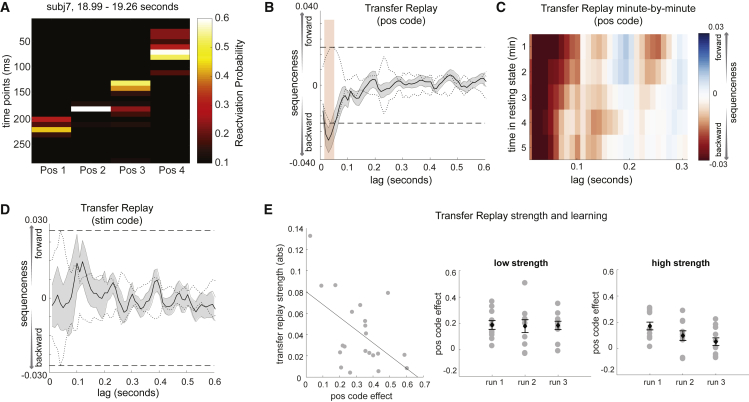
“Transfer Replay” of Position Code before Exposure to New Stimuli On day 2, participants had a rest period in the scanner before exposure to the novel objects. During this rest period, we observed reverse sequences of *pos* code reactivations (i.e., position 4, position 3, position 2, position 1). (A) Example of a reverse sequence event made up of *pos* codes. (B) Statistically, there was strong evidence across subjects for reverse sequences of *pos* codes. (C) This reverse sequenceness was stable for all 5 min of rest. (D) As a sanity check, we also looked for replay of the *stim* code in the first rest period, which should not be possible because the stimuli have not yet been seen. We found no evidence of such activity. (E) Between subjects, the strength of preplay (from A) was negatively correlated with the degree of *pos* code activation during applied learning (from Figure 5A, red trace). Subjects with low preplay (by median split) expressed the position code strongly throughout learning. Conversely, subjects with high preplay had a steep falloff in position code activation across learning. Each gray dot indicates an individual subject, with error bars representing the standard error of the mean across subjects. See also Figures S4 and S5.

For this analysis, we took advantage of the first resting period at the beginning of the MEG scan, before participants experienced day 2 objects. Using trained models of position codes, we performed the same sequenceness analysis as previously described, using the transition matrix position 1 → position 2 → position 3 → position 4. Examples of sequential reactivations of position codes during the first resting period are shown in Figure 6A. Statistically, we found significant reverse sequenceness, peaked at a 30-ms position-to-position lag (n = 21, β = −0.036 ± 0.008), exceeding a permutation threshold from 20- to 60-ms time lag (Figure 6B). We refer to this phenomenon as “transfer replay” because it links day 2 objects to previous experiences, as well as to avoid confusion with the complex preplay literature.

Transfer replay appeared within the first min of rest (Wilcoxon signed-rank test, p = 0.011), remained stable for all 5 min of rest (second min, p = 0.011; third min, p = 0.0006; fourth min, p = 0.007; fifth min, p = 0.0005; Figure 6C), and was evident for most participants (Figure S4E; examples of individual sequences in Figure S4F, right). As a sanity check, we also tested for replay of stimulus codes during the first resting period, which should not be possible since stimuli had not yet been experienced. Reassuringly, we found no evidence for such sequenceness (Figure 6D).

If transfer replay constitutes “rehearsal” of structural knowledge, we might ask whether individuals with stronger transfer replay are quicker to apply structural knowledge to new objects. Consequently, we measured the relationship between transfer replay strength and position code reactivation during each run of learning. Participants with greater transfer replay had less overall position code reactivation, and this effect was driven by a decrease in position code reactivation over learning in participants with high but not low transfer replay (p = 0.007 interaction term in regression; Figure 6E). We speculate that this reflects individuals with high transfer replay rapidly learning a position-to-stimulus mapping.

### Power Increase in Sharp-Wave Ripple Frequencies around Replay Events

**Figure 7:**
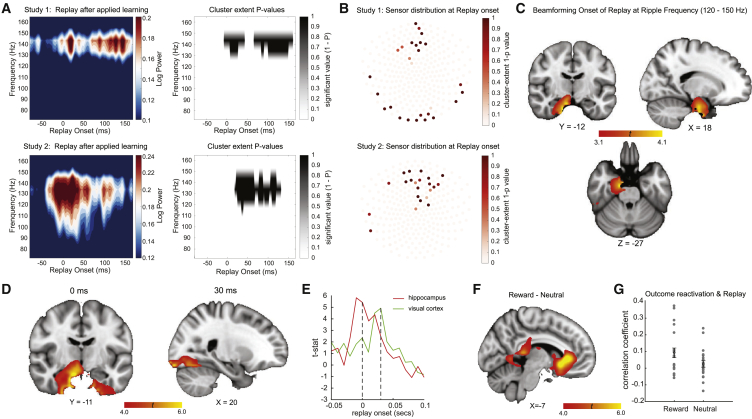
Replay Coincides with Ripple-Band Power, which Source Localizes around the Hippocampus (A) Left: in the ripple frequency band (120−150 Hz), there was a significant power increase (averaged across all sensors) at the onset of replay, compared to the pre-replay baseline (100−50 ms before replay). Right: a cluster-based permutation test (cluster forming threshold t > 3.1, number of permutations = 5000) identified a significant cluster around 140 Hz. This effect replicated across study 1 and study 2. Note that replay events were excluded if there was another replay event in the baseline period (see Figure S7). (B) Sensor distribution of p values (plotted as 1-p) for ripple-band power increase at the replay onset. We found similar sensor patterns in study 1 and study 2. (C) Combining data from two studies, source localization of ripple-band power at the replay onset revealed significant hippocampal activation (peak Montreal Neurological Institute [MNI] coordinate: X = 18, Y = −12, Z = −27). (D) We also contrasted broadband power at replay onset times against the pre-replay baseline. This contrast also found activity in the medial temporal lobe that encompassed the bilateral hippocampus (peak MNI coordinate: X = 16, Y = −11, Z = −21). When performing the same contrast but using onsets 30 ms after replay, we found the visual cortex (peak MNI coordinate: X = 20, Y = −97, Z = −13). The source image was thresholded at *t* > 4.0 (uncorrected) for display purposes. Both the hippocampus (at 0 ms) and visual cortex (at 30 ms) survived whole-brain multiple comparison correction based on a non-parametric permutation test. (E) The time course of the hippocampus at its peak MNI coordinate is shown in red, while the visual cortex at its peak MNI coordinate is shown in green. (F) We also examined the contrast of rewarded sequence replay onset against unrewarded sequence replay onset in study 1. In this contrast, we found vmPFC activation. (G) We trained decoding models to detect representations of reward outcomes and neutral outcomes. Spontaneous reactivation of reward outcome representations was correlated with the replay of the rewarded sequence. There was no correlation between the reactivation of the neutral outcome and the replay of the neutral sequence. Error bars represent SEM across subjects. See also Figure S7.

**Figure S7:**
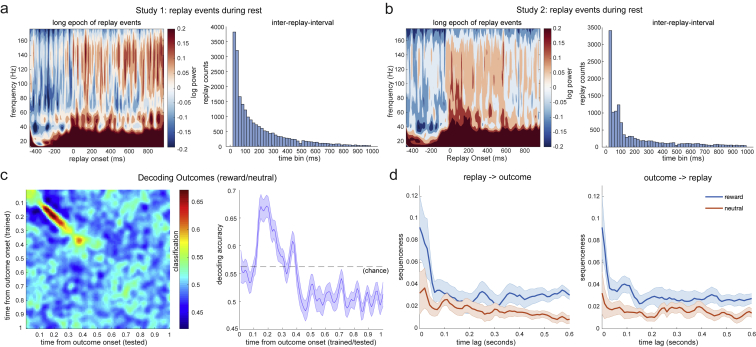
Replay Events in Long Epoch, Inter-Replay-Interval, and Outcome Reactivation, Related to Figure 7 a, b, Time-frequency maps shown for an extended epoch after replay events in study 1(a) and 2 (b). 0ms is the onset of replay events. Notably these are only replay events which are not preceded by other replay events in the previous 100ms. Note that the increase in ripple-band and low frequency power extends for several hundred milliseconds. This can be explained by the fact that replay events occur in clusters. Histograms show the inter-replay intervals across all replay events in all subjects in study 1(a) and study 2(b). The modal replay onset time is immediately following the previous replay event. The heavy-tail of the distribution indicates that there are also periods with no replay events. c, To find the peak time point to train outcome decoders in the value learning phase from Study 1, we trained classifiers on every time point relative to the onset of outcome, and tested at every time bin relative to the same onsets. Each cell of this grid shows cross-validated (leave-one-out) prediction accuracy. We found the peak decoding accuracy was around 200 ms after the stimuli onset. d, The reward outcome reactivated at the same time as the onset of replay of the rewarded sequence during rest, while no such relationship was observed for neutral outcome and replay of neutral sequence.

In rodents, spontaneous offline replay events co-occur with bursts of high-frequency (120−200Hz) local field potential power known as sharp-wave ripples (SWRs) (Buzsáki, 2015). To see if we could detect a similar phenomenon in humans, we performed time-frequency analysis in both studies (Figure 7A; see also Figures S7A and S7B for time-frequency analysis in longer epoch and inter-replay-intervals from both studies). We evaluated frequencies up to 150 Hz, the maximum allowed by our data acquisition methods.

Individual replay events were defined as moments with a high probability of a stimulus reactivation that were also followed 40 ms later by a high probability of reactivation of the next stimulus in the sequence (see Methods for details). At the onset of replay, we found a power increase at 120−150 Hz, compared to a baseline period 100−50 ms before replay events. This increase lasted approximately 100 ms and was significant in both studies (Figure 7A; cluster-based permutation test with cluster forming threshold *t* > 3.1 and number of permutations = 5000).

To examine this effect in sensor space, we averaged across power changes in the frequency range 120−150 Hz and then ran permutation-based analysis on the sensors^∗^time map (cluster forming threshold *t* > 3.1, 5000 permutations). The pattern of sensors with increased SWR-band power at replay onset was similar in both studies (Figure 7B) and strongly resembled the distribution over sensors that corresponds to intracranially recorded hippocampal local field potentials (LFPs) (Dalal et al., 2013).

### Source Localization of Replay

In general, source-localizing neural activity measured using MEG should be treated with caution due to inaccuracies in the forward model and the priors used to invert it (cf. Hillebrand and Barnes, 2002). With this caveat in mind, we asked about the likely anatomical sources of replay using resting state data combined from both studies.

We epoched the data using replay onset events and beamformed power in different frequency bands into source space (see Method Details for details). When we considered either ripple frequencies alone (120−150 Hz; Figure 7C) or broadband power across all frequencies (0−150 Hz; Figure 7D, left); power increases at the onset of replay events were associated with sources in the medial temporal lobe. Notably, broadband power increases 30 ms after replay events were associated with sources in visual cortical regions (Figure 7D, right). Each of these power increases survived statistical thresholding by a permutation test (p < 0.05 corrected; see Method Details). For display purposes, we extracted the peak coordinate from the hippocampus and visual cortex, respectively, and plotted the respective time courses of their broadband power (Figure 7E). In future experiments, it would be intriguing to test the idea that relational knowledge embedded in medial temporal areas orchestrates the replay of sensory representations.

To localize the neural difference between the replay of rewarded and neutral sequences in study 1, we contrasted the onset of the rewarding sequence against the onset of the neutral sequence. We found activation in the ventral medial prefrontal cortex (vmPFC), extending to the ventral striatum (Figure 7F). Given vmPFC is known to encode value, we tested whether reward representations were associated with the rewarded sequence during rest. As a supplemental analysis, we separately trained a classifier, using data from the times of outcome deliveries, to distinguish reward from non-reward outcomes (Figure S7C). We then applied this classifier to the second resting period in study 1. We found that reward outcome representations were coincident with the onset of a rewarded sequence (two-tail one sample t test against zero, t (20) = 3.20, p = 0.005), with no such relationship between a neutral outcome representation and onset of the neutral sequence (t (20) = 1.17, p = 0.26) (Figures 7G and S7D). We speculate that reward representation in vmPFC may play a role in initiating the replay of a rewarded sequence.

## Discussion

At rest, the hippocampal-entorhinal system and neocortex spontaneously play out rapid-fire sequences of real and fictive spatial trajectories, decoupled from current sensory inputs (Foster, 2017). Here, we first bolster preliminary evidence that such replay also exists in non-spatial domains and can be measured non-invasively in humans. We show that fictive replay does not merely stitch together experienced sub-trajectories but constructs entirely novel sequences in an inferred order determined by abstract structural knowledge. Finally, we observe that each replayed sequence comprises not only representations of the stimuli in the sequence, but also representations of the abstract structure. We propose that this abstract replay is a mechanism for generalizing structural knowledge to new experiences.

### Non-spatial Replay

Spatial replay is a remarkable neural mechanism that bridges our understanding of circuit mechanisms and computational functions (Buzsáki and Moser, 2013, Foster, 2017). If replay is a ubiquitous feature of brain activity that extends beyond the spatial domain, it may contribute to learning and inference that relies on arbitrary conceptual knowledge. Rapid spontaneous sequences of non-spatial representations have previously been observed in humans (Kurth-Nelson et al., 2016). Three features of the current study bring these measurements closer to rodent hippocampal replay: the sequences are present during rest (Foster and Wilson, 2006), they reverse direction after rewards (Ambrose et al., 2016), and they coincide with an increase in source-localized hippocampal power in ripple frequency (Buzsáki, 2015). Together with the degree of time-compression, these findings provide strong parallels and convergence with rodent replay events seen during SWRs.

### Factorization and Replay of Inferred Sequences

In rodents, there is evidence for replay of never-before-experienced sequences that are consistent with the geometry of a spatial map (Gupta et al., 2010, Ólafsdóttir et al., 2015). Here, we ask whether, like spatial geometry, learned arbitrary structures impose constraints on replay. Participants first learned an arbitrary unscrambling rule that defined a sequence over a set of objects. After experiencing a novel set of objects, replay immediately sequenced the objects according to this rule rather than to the order of the experience. This can be viewed as “meta-learning” (Harlow, 1949, Shin et al., 2017, Wang et al., 2018), insomuch as previous learning facilitated rapid integration of new knowledge.

Generalization of learned structures to new experiences may be facilitated by representing structural information in a format that is independent from its sensory consequences, as do grid cells in spatial experiments. Factorized representations are powerful, as they allow components to be recombined in many more ways than were experienced (Behrens et al., 2018, Bernardi et al., 2018, Higgins et al., 2017b). The ability to recombine knowledge during replay may allow disparate observations to be compiled into meaningful episodes online (Botvinick and Toussaint, 2012, Pezzulo et al., 2014). Equally importantly, it may also allow simulated experiences to drive cortical learning offline. Using time to bind new combinations (Jensen et al., 2014) is attractive, as it avoids the requirement for a combinatorically large representational space (see also Kumaran and McClelland 2012).

In rodents, disruption of replay containing SWR events degrades subsequent spatial memory performance (Girardeau et al., 2009, Jadhav et al., 2012), implying a causal role for spatial replay. However, we cannot be certain the same causal role exists for the factorized replay described here. Future experiments using online detection and disruption of replay events will be needed.

### Anatomy of Replay

Although it is difficult to make confident anatomical statements with MEG, an advantage is simultaneous recording from the whole brain. Given the weight maps of our object classifiers, it is most likely that the spontaneous sequences we detected were sequences of neocortical representations. At the same time as these sequences appeared, there was also a transient increase in power around 140 Hz, which source localized to the medial temporal lobe. These observations are consistent with the idea that replay may be coordinated between the hippocampus and neocortical areas (Ji and Wilson, 2007). Moreover, by taking whole-brain measurements, we were able to make an observation that has not been reported in rodents. The vmPFC activated 10 ms prior to reward-associated reverse replay events, hinting that such events might be instructed by the reward system. These results commend simultaneous recordings in a rodent’s prelimbic cortex and hippocampus during rest.

### Transfer Replay

On day 2, abstract representations of position replayed after learning and also played out before applied learning (in the first rest period). This raises an interesting analogy to what is termed “preplay” in rodent research (Dragoi and Tonegawa, 2011). In our human data, these same position codes were later tied to new stimuli during the learning phase and during post-learning replay on day 2. Furthermore, the degree to which they played out before learning predicted the effects seen during applied learning. Therefore, it is plausible that transfer replay is used to support learning about new stimuli. In this spirit, transfer replay bears resemblance to preplay reported in a rodent’s hippocampus. Indeed, it is also possible that preplay sequences reported in a rodent’s hippocampus may have been learned through the experience of other sequences. However, we cannot determine if the pre-experience sequences in our data indeed reflect learned structure or a preconfigured neural dynamic, as previously suggested in the literature (Dragoi and Tonegawa, 2013b, Grosmark and Buzsáki, 2016). An interesting possibility is the position codes might not only be abstracted over different sets of objects within this particular task, but also, in fact, be common to other transitively ordered sequences (Kornysheva et al., 2019). In rodents, preplay has been suggested to reflect the knowledge of a common structure of space (Dragoi and Tonegawa, 2011, Dragoi and Tonegawa, 2013a, Liu et al., 2018). Similarly, tasks that involve inference on linear structures may benefit from representations of their shared underlying statistics (Behrens et al., 2018, Luyckx et al., 2019).

### Conclusions

The ability to measure replay in humans opens new opportunities to investigate its organization across the whole brain. Our data suggest powerful computational efficiencies that may facilitate inferences and generalizations in a broad array of cognitive tasks.

## STAR★Methods

### Key Resources Table

**Table:** 

REAGENT or RESOURCE	SOURCE	IDENTIFIER
**Deposited Data**		

MEG data	This paper	N/A

**Software and Algorithms**		

MATLAB	MATLAB	https://www.mathworks.com/products/matlab.html
Custom code and algorithms	This paper	https://github.com/YunzheLiu/FactorizedReplay

**Other**		

Human healthy participants recruited from local area	This paper	N/A
Neural recordings and amplifier	**Whole Brain CTF MEG 275 System**	https://www.ctf.com/

### Lead Contact and Materials Availability

Further information and requests for resources and reagents should be directed to and will be fulfilled by the Lead Contact, Yunzhe Liu (yunzhe.liu.16@ucl.ac.uk).

### Experimental Model and Subject Details

#### Participants

Twenty-five participants (aged 19-34, mean age 24.89) participated in the first study. Eleven were male, and one was left-handed. Three were excluded before the start of analysis because of large movement (> 20 mm) or myographic artifacts. Data from another participant was unusable due to missing triggers, leaving 21 participants in total for further analyses in Study 1. A separate cohort of twenty-six participants (aged 19-34, mean age 25.48) participated in the second study. Ten were male and two were left-handed. Two of these were excluded before the start of analysis due to movement-related (> 20 mm) or myographic artifacts. Another two participants were excluded due to failure of understanding of the task, leaving 22 participants in total for further analyses in Study 2. All participants were extensively trained the day before the MEG task and had a mean accuracy of at least 80% on probe trials of the sequence representation (Figure S1). In all analyses, no sex or gender identity related difference was found.

All participants were recruited from the UCL Institute of Cognitive Neuroscience subject pool, had normal or corrected-to-normal vision, no history of psychiatric or neurological disorders, and had provided written informed consent prior to the start of the experiment, which was approved by the Research Ethics Committee at University College London (UK), under ethics number 9929/002.

### Method Details

#### Task

##### Study 1

In the first study, we exploited a revised sensory preconditioning paradigm (Figure 1A). There were eight visually distinct objects. Four objects constituted one single sequence, providing for two distinct sequences (i.e., A- > B- > C- > D; A’- > B’- > C’- > D’). Participants were initially presented with objects shuffled in order (e.g., C- > D; B- > C; A- > B); and were subsequently required to rearrange them in a correct sequential order (e.g., A- > B- > C- > D) without ever having experienced this full trajectory. Participants were trained a day before scanning with different stimuli, meaning they were trained on the actual structure of the task. Then, on the second day, participants underwent MEG scanning while performing a similar task but now with different stimuli. The task was implemented in MATLAB (MathWorks) using Cogent (Wellcome Trust Centre for Neuroimaging, University College London).

On Day 1, participants went through four runs of training. Each run consisted of three phases, where each phase was repeated three times. Participants viewed eight distinct pictures. The pictures appeared sequentially, but participants were aware that this order of appearance was a scrambled version of a different sequential order which would be crucial for obtaining reward later. The underlying true order contained two sequences. However, in the sequence presented to subjects, each transition in a true sequence was presented together with transitions from the other true sequence, and the learning of each transition occurred in a separate stage. For example, the true sequences WXYZ and W’X’Y’Z’ might be presented in these three stages: [YZ, Y’Z’], [XY, X’Y’], [WX, W’X’]. The interval within a pair was 300 ms and the interval between pairs was 900 ms. Before viewing the scrambled sequences, participants were carefully instructed on the rule that unscrambled the six visual transitions into the two true sequences. For example, if a participant observed [YZ Y’Z’], they could deduce that YZ is part of one true sequence and Y’Z’ is part of the other. After each run of visual presentation, participants were probed on the true order of the sequences. On each probe trial, the probe stimulus was presented on the center of the screen and two other stimuli were presented below. One of the two stimuli was always selected from later in the same true sequence as the probe, and the other stimulus was randomly selected either from before in the same true sequence or from any position in the different sequence. Participants were asked to identify this stimulus. For example, if X was the probe stimulus, and the two alternatives were W and Z, the correct answer would be Z. Participants were only admitted to the Day 2 MEG experiment if they achieved an average accuracy of at least 80% on probe trials. To prevent learning during probe trials, no feedback was given.

On Day 2, in the MEG scanner, participants experienced a new set of pictures. These pictures were first presented in a randomized order, as a functional localizer, in order to train classification models. Before presentation of each image, a word describing the image appeared in text for a variable duration of 1.5 to 3 s, followed immediately by the picture itself. The use of a semantic cue was borrowed from Kurth-Nelson et al. (2016). In piloting, the semantic cue gave the best decoding at the interesting 200 ms time point. We speculate that the semantic cue might encourage prediction mechanisms or favor a richer representation of the stimuli that might facilitate detection in replays–but to our knowledge this has never been directly tested. On 20% of trials, the object was upside-down. To maintain attention, participants were instructed to press one button if the object was correct-side-up, and a different button if it was upside-down. Once the participant pressed a button, the object was replaced with a green fixation cross if the response was correct and a red cross if the response was incorrect. This was followed by a variable length inter-trial interval (ITI) of 700 to 1,700 ms. There were two sessions, each session included 120 trials, with 24 correct side-up presentations of each visual object in total. Only correct-side-up presentations were used for classifier training. The trial order was randomized for each participant and visual object and state mapping was randomized across participants.

Next, participants were presented with Day 2′s pictures in a non-random but scrambled order. We call this Applied Learning. As on Day 1, this scrambled order was a permutation of two “true” sequences. Unlike Day 1’s true sequences, Day 2′s true sequences were never seen. But, because the permutation that mapped true sequences to scrambled sequences was the same across Day 1 and Day 2, this enables subject to infer Day 2′s true sequences. There were three blocks of Applied Learning. Each block had three phases, and each phase presented two pairwise associations, one from each unscrambled sequence. In each phase, objects from the two associations were presented consecutively, each stimulus was presented for 900 ms, followed by an inter-stimulus interval (ISI) of 900 ms, then followed by the other pairwise association. Each phase was repeated three times, then followed by the next phase. Each block was followed by multiple choice questions designed to probe whether participants had correctly inferred the true sequences. At each probe trial, the probe stimulus appeared for 5 s during which participants need to think about which object followed the probe stimulus in the true sequence, and then selected the correct stimulus from two alternatives. No feedback was provided. There was a 33% possibility that the wrong answer came from the same sequence but was preceding instead of following the probe stimuli. This setup was designed to encourage participants to form sequential rather than clustering representations (i.e., which sequence does this object belong to).

After the Applied Learning, participants had a 5 mins rest period, during which they were not required to perform any task. After the 5 min rest period, participants were then taught that the end of one sequence led to monetary reward, while the end of the other did not, in a deterministic way. In each trial, participants saw the object of each end of the sequence (i.e., D or D’) for 900 ms, followed by an ISI of 3 s, and then either received a reward (image of a one-pound sterling coin) or no-reward (blue square) outcome for 2 s, followed by an ITI of 3 s. Objects appeared 9 times, for a total of 18 trials. Participants were required to press one button for the reward, and a different button for non-reward. Pressing the correct button to ‘pick up’ the coin led to a payout of this money at the end of the experiment (divided by a constant factor of ten), and participants were pre-informed of this. After value learning, participants had another rest period, for 5 mins, without any task demands.

As a final assignment, participants were asked to perform a model-based decision-making task. Here they had to determine whether presented stimuli led to reward or not. In each trial, an object was presented on the screen for 2 s, and participants were required to make their choice within this 2 s time window, followed by ITI of 3 s. Each stimulus was repeated 5 times such that there were 40 trials in total, 20 for each sequence. The trial order was fully randomized with a constraint that the same stimulus would not appear consecutively. No feedback was provided after a response so as to eliminate learning at this stage. After the task, participants were required to write down two sequences in the correct order. All participants were 100% correct, suggesting they maintained a task structure representation until the end of the task.

##### Study 2

In Study 1, the scrambling rule was such that scrambled sequences shared all individual pairwise transitions with unscrambled sequences. This admitted a possible interpretation of unscrambled sequence replay as arising from a simple Hebbian mechanism without any transfer of knowledge from Day 1 to Day 2. We designed Study 2 to rule out this interpretation (Figure 4A).

The overall methods for Study 2 were very similar to Study 1. However, the unscrambling rule differed. In Study 2, stimuli were presented in an order that did not contain any pairwise transitions of the true sequences. The mapping between visual order and structure order was completely randomized for each participant, subject to the constraint that stimuli alternated between the two true sequences. Another difference from Study 1 was that stimuli were presented in two stages instead of three.

On Day 1, before training, participants were explicitly instructed on the mapping between the order of visual presentation and the two true sequences. For example, stimuli might be presented in the following two stages: [Z’ X Y’ Y], [Z W’ W X’]. The participant with this scrambled order was carefully instructed that this order defines two true sequences: WXYZ and W’X’Y’Z’.

Participants underwent four runs of training, with two stages in each run. Each stage was presented three times, with an equal time gap (500 ms) between each stimulus. After each run, they were quizzed about the true order, without feedback, in a similar fashion as Study 1. To progress to Day 2, participants were required to reach at least 80% average accuracy.

On Day 2, participants were required to perform a MEG experiment with a different set of stimuli, but under the same structure. First, participants were allowed a 5-min rest period at the beginning of MEG experiment before any experience of the new stimuli. This was similar to the setting of Dragoi and Tonegawa (2011), where “preplay” sequences were measured before experience with a new spatial environment. Here, we aimed to detect sequential replay of abstract structural code that they learnt the day before, we called it “transferred replay.”

After a resting period, participants performed a functional localizer task, as in Study 1. This was then used to train classification models of stimuli. Note, the functional localizer task preceded Applied Learning, so participants had no structure information at the time of the localizer. These decoding models therefore capture a sensory level in neural representations of stimuli (i.e., stimulus code).

Participants were then presented with the stimuli and required to unscramble the “visual sequence” into a correct order, i.e., the “unscrambled sequence” based on the template they learnt the day before. There were two phases in each block, each phase comprised four images with each stimulus presented for 900 ms, followed by an inter-stimulus interval (ISI) of 900 ms. Each phase was repeated three times, then followed by the next phase. There were three blocks in total, each block followed by multiple choice questions without feedback, similar to the first study. However, there were two differences in Study 2 compared to Study 1. First, in Study 1, all three images appeared on the screen together. In Study 2, the probe stimulus appeared alone for 5 s, and then the two candidate successor images appeared. Second, in Study 1, participants had 5 s to make a choice. In Study 2, this was reduced to 600 ms. These changes were intended to limit further potential associative learning when the stimuli were presented together.

After the Applied Learning, participants were given a rest for 5 mins again. We were interested 1) to replicate our findings in the first study, i.e., replay stitched together independent events into a sequence that is confined by the relational structure; 2) to measure abstract representations of structure and their relationships to representations of concrete objects.

The session concluded with a task where stimuli were presented in random order, and participants were asked to identify either which true sequence the stimulus belonged to, or which position the stimulus occupied within its true sequence. These two types of trials were divided into blocks and the order of the blocks was counterbalanced across participants. In each trial, the object was presented on screen for 2 s, and participants were required to respond with its sequence or its position within these 2 s. The inter-trial interval was 3 s. Each stimulus repeated 10 times in each block with a total of 160 trials: 80 for position testing (20 trials for each position) and 80 for sequence testing (40 trials for each sequence). The trial order was fully randomized with a constraint that the same stimulus would not appear consecutively. No feedback was provided. After the task, as in the first study, participants were required to write down the unscrambled sequences in the right order, and all participants were 100% correct.

### Quantification and Statistical Analysis

#### MEG Acquisition and Pre-processing

The same procedures for MEG acquisition and preprocessing were applied to both studies. MEG was recorded continuously at 600 samples/second using a whole-head 275-channel axial gradiometer system (CTF Omega, VSM MedTech), while participants sat upright inside the scanner. Participants made responses on a button box using four fingers as they found most comfortable.

The data were resampled from 600 to 100 Hz to conserve processing time and improve signal to noise ratio. All data were then high-pass filtered at 0.5 Hz using a first-order IIR filter to remove slow drift. After that, the raw MEG data were visually inspected, and excessively noisy segments and sensors were removed before independent component analysis (ICA). An ICA (FastICA, http://research.ics.aalto.fi/ica/fastica) was used to decompose the sensor data for each session into 150 temporally independent components and associated sensor topographies. Artifact components were classified by combined inspection of the spatial topography, time course, kurtosis of the time course and frequency spectrum for all components. Eye-blink artifacts exhibited high kurtosis (> 20), a repeated pattern in the time course and consistent spatial topographies. Mains interference had extremely low kurtosis and a frequency spectrum dominated by 50 Hz line noise. Artifacts were then rejected by subtracting them out of the data. All subsequent analyses were performed directly on the filtered, cleaned MEG signal, in units of femtotesla.

#### MEG Analysis

##### Multivariate Decoding Analysis

Lasso-regularized logistic regression models were trained separately for sensory representations (i.e., “stimulus codes”) and structure representations (i.e., “position codes” and “sequence codes”). Only the sensors that were not rejected across all scanning sessions in the preprocessing step were used to train the decoding models. A trained model *k* consisted of a single vector with length of good sensors n + 1: slope coefficients for each of the good sensors together with an intercept coefficient. In both studies, decoding models were trained on MEG data elicited by direct presentations of the visual objects.

For each object we trained one binomial classifier. Positive examples for the classifier were trials on which that object was presented. Negative examples consisted of two kinds of data: trials when another object was presented, and data from the fixation period before the semantic pre-cue appeared. The null data were included to reduce the correlation between different classifiers–so it was possible for all classifiers to report low probabilities simultaneously in rest data. Prediction accuracy was estimated by treating the highest probability output among all classifiers as the predicted object. Sensor distributions of beta estimates and prediction performance of classifiers trained on 200 ms on left-out trials in functional localizer task and subsequent applied learning task from both studies are shown in Figure S3.

The design of Study 2 enabled us to dissociate between the neural representation of sensory and structure level of stimuli. To check whether these classifiers learned abstract information about position and sequence, rather than relying on coincidental sensory features of the stimuli, we used a special cross-validation approach. Instead of holding out individual trials at random, we held out all the trials of one randomly selected object. This meant that a classifier that focused on sensory features would result in below-chance accuracy. To perform above chance, the classifier must identify features that represent the structural information (position or sequence). Using a permutation-based threshold, which corrected for multiple comparisons across time, we found that cross-validated decoding accuracy exceeded chance for both position and sequence code classifiers (Figures S5B and S5C).

Because we did not randomize button assignments in the position and sequence judgement task, we were concerned about a possible motor confound. Therefore, we used position judgement trials to train sequence code classifiers, and we used sequence judgement trials to train position code classifiers. In cross-validation on training data, accuracy peaked at 300 ms post stimulus onset for the position code, and at 150 ms post stimulus onset for the sequence code. One participant was excluded from position code analysis due to below-chance position decoding. Two participants were excluded from sequence code analysis due to below-chance sequence decoding or missing data.

To explore which MEG sensors contained the most information about object-level and abstract-level representations of stimuli, we repeatedly performed the same cross-validation analysis described above, but each time using a different random subset of 50 sensors. On each iteration, we found a classification accuracy. After performing this analysis 2000 times with different random subsets, we averaged all the accuracies that each sensor participated in, to obtain an approximation of that sensor’s contribution to predicting the labels. We observed that the *stim* code mainly involved features around occipital sensors (Figure S5D), while the *pos* and *seq* codes were more focused in posterior temporal areas (Figures S5E and S5F).

##### Sequenceness Measure

The code for this analysis is supplied along with a readme as supplementary material. The analysis is described in detail below along with an accompanying explanatory figure (Figure S1).

The decoding models described above allowed us to measure spontaneous reactivation of task-related representations during resting periods. We next defined a ‘sequenceness’ measure, which describes the degree to which these representations were reactivated in a well-defined sequential order.

Here we describe how this measure was computed for stimulus sequences (the computation was nearly identical for position code sequences). First, we applied each of the eight stimulus decoding models to the resting MEG data. This yielded eight timeseries of reactivation probabilities, each with length N, where N is the number of time samples in the resting data (Figure S1A).

We then used a linear model to ask whether particular sequences of stimulus activations appeared above chance in these timeseries. For each stimulus i, at each possible time lag Δt, we estimated a separate linear model:

$$Y_{i}=X\left(\Delta t\right)\beta \left(\Delta t\right)$$

The predictors X(Δt) were time-lagged copies of the eight reactivation timeseries, along with nuisance regressors (described below). The model predicted $Y_{i}$, the reactivation of stimulus i. The linear model had N rows, with each row a time sample. We estimated $\beta_{i}\left(\Delta t\right)$, a vector of coefficients that described the degree to which stimulus i’s reactivation was predicted by activation of each other stimulus at time lag Δt. By repeating this procedure for each stimulus i, we obtained β(Δt), an 8x8 matrix that can be viewed as an empirical transition matrix between the eight stimuli at lag Δt (Figure S1B).

Specifically:

$$Y_{i}=\sum_{j=1}^{s}X_{j}\left(\Delta t\right)\beta_{ij}\left(\Delta t\right)$$

Where$\;X_{j}\left(\Delta t\right)$are time-lagged copied of$\;Y_{j},$ s is the number of states, and therefore:

$$Y_{i}\left(t\right)=\sum_{j=1}^{s}Y_{j}\left(t-\Delta t\right)\beta_{ij}\left(\Delta t\right)$$

The matrixβ(Δt)is obtained by solving the following set of equations for each stimulusi, up to state s.

$$Y_{i=1}\left(t\right)=\sum_{j=1}^{s}Y_{j}\left(t-\Delta t\right)\beta_{ij}\left(\Delta t\right)$$

$$Y_{i=2}\left(t\right)=\sum_{j=1}^{s}Y_{j}\left(t-\Delta t\right)\beta_{ij}\left(\Delta t\right)$$

$$Y_{i=s}\left(t\right)=\sum_{j=1}^{s}Y_{j}\left(t-\Delta t\right)\beta_{ij}\left(\Delta t\right)$$

We next asked whether the β(Δt) was consistent with a specified 8x8 transition matrix by taking the Frobenius inner product between these two matrices (the sum of element-wise products of the two matrices). This resulted in a single number $Z_{\Delta t}$, which pertained to lag Δt (Figure S1C). Finally, sequenceness was defined as $Z_{f\Delta t}-Z_{b\Delta t}$. We used the difference between correlations in the forward $\left(Z_{f\Delta t}\right)$ and backward $\left(Z_{b\Delta t}\right)$ direction in order to remove common autocorrelation which would otherwise add significant variance.

This analysis is very similar to the sliding correlation analyses described in Kurth-Nelson et al. (2016), with the important exception that in computing a correlation between stimuli i and j, our method controls for other covariates. These covariates include the activation of other stimuli *k*≠i*,j* as well as nuisance regressors. In our data we observed a strong alpha rhythm, consistent with the fact that most participants closed their eyes during the resting period. A strong alpha rhythm can contaminate the sequenceness measure, causing a drop in sensitivity. We therefore included as nuisance regressors additional time-lagged copies of the reactivation timeseries at lags Δt+100ms, Δt+200ms, … up to Δt+600ms, to control for 10 Hz oscillation. These nuisance regressors will capture any neural patterns that repeat with 10Hz frequency. This variance will therefore not contaminate the regressors of interest. We also added a constant term to the design matrix.

In the current study, the transition matrix was defined as either the rule-defined stimulus order (i.e., “unscrambled sequence”) or the experienced visual order of stimuli (i.e., “scrambled sequence”).

For statistical testing, we used nonparametric tests involving all possible permutations of the stimulus labels (equivalent to permuting together the rows and columns of the transition matrices used to calculate sequenceness). Stimulus identities were exchangeable under the null hypothesis that there are no systematic relationships between the reactivations of different stimuli. To further ensure the results were not overfit to the regularization parameter of the logistic regression, all results were obtained in cross-validation (by leave-one-subject-out) over this parameter.

We also computed the extent to which neural sequences followed longer steps (length-3 or length-4) with the same stimulus-to-stimulus time lag, while controlling for evidence of shorter length. By controlling for shorter lengths, we avoid a possibility of false positives arising out of strong evidence for shorter length. The method is largely the same as the GLM approach described above, with the addition of shorter length transitions in the design matrix as confounding regressors. For example, if we look for evidence of A- > B- > C at 50 ms time lag, we regress stimulus decoding vector A with time lag 100 ms onto C while controlling for evidence of stimulus decoding vector B reactivated at time lag 50 ms. This process can be generalized to any number of steps. In the current study, the maximum possible length was 4. Note that this method does not allow us to rule out the possibility that the data contained both AB and A_C, but not ABC; however, we do not think this is likely.

In both studies, the decoding models used to evaluate sequenceness of stimulus representations were trained on functional localizer data taken from 200 ms following stimulus onset. The 200 ms time point was used for consistency with Kurth-Nelson et al. (2016). In that study it was originally selected based on the observation that retrieval of this 200 ms slice of the evoked representation was linked to model-based reasoning (Kurth-Nelson et al., 2015). For completeness, in the present work we also performed sequenceness analysis using *different* time points (relative to stimulus onset in the functional localizer) to train the decoding models. In both Study 1 and Study 2, we found that evidence for sequenceness fell off rapidly when training on times either before or after 200 ms (Figures S6A−S6C). We then re-analyzed data from Kurth-Nelson et al. (2016) and found a very similar pattern (Figure S6D). It is intriguing that resting data consistently contains sequences of only this particular time-slice of the evoked representation.

##### Identifying Replay Onsets

Replay onsets were defined as moments when a strong reactivation of a stimulus was followed by a strong reactivation of the next stimulus in the sequence. Specifically, we first found the stimulus-to-stimulus time lag Δt at which there was maximum evidence for sequenceness, time shifted the reactivation matrix X up to this time lag Δt, obtained X(Δt). We then multiplied X by the transition matrix P, corresponding to the unscrambled sequences: X×P. Next, we elementwise multiplied X(Δt) by X×P. The resulting matrix had a column for each stimulus, and a row for each time point in the resting data. We then summed over columns to obtain a long vector R, which each element indicating the strength of replay at a given moment in time. Finally, we thresholded R at its 95th percentile. We also imposed a constraint that a replay onset has 100 ms of replay-free time preceding them.

Specifically:

Proj=X(Δt)

Matrix Proj is obtained by time shifting the reactivation matrix X to time lag Δt.

Orig=X×P

Matrix Orig is obtained by matrix multiplication between reactivation matrix X and transition matrix P.

$$R_{t}=\sum_{i}^{s}Orig_{ti}*Proj_{ti}$$

Vector R is obtained by elementwise multiplication between matrix Orig and Proj, and then sum over columns.

#### MEG Source Reconstruction

All source reconstruction was performed in SPM12 and FieldTrip. Forward models were generated on the basis of a single shell using superposition of basis functions that approximately corresponded to the plane tangential to the MEG sensor array.

Linearly constrained minimum variance beamforming (Van Veen et al., 1997) was used to reconstruct the epoched MEG data to a grid in MNI space, sampled with a grid step of 5 mm. The sensor covariance matrix for beamforming was estimated using data in either broadband power across all frequencies or restricted to ripple frequency (120-150 Hz). The baseline activity was the mean neural activity averaged over −100 ms to −50 ms relative to replay onset. All non-artifactual trials were baseline corrected at source level. We looked at the main effect of the initialization of replay. To explore reward and neutral sequences in Study 1, we applied the above approach separately for reward and neutral sequence and contrasted reward versus neutral trials at source level.

Non-parametric permutation tests were performed on the volume of interest to compute the multiple comparison (whole-brain corrected) P values of clusters above 10 voxels, with the null distribution for this cluster size being computed using permutations (n = 5000 permutations).

### Data and Code Availability

#### Data availability

Data used to generate the findings of this study will be freely available upon request (subject to participant consent) to the Lead Contact.

#### Code availability

Custom computer code used to generate the findings of this study will be made available upon request to the Lead Contact. Simulation code can be found in https://github.com/YunzheLiu/FactorizedReplay

## References

[bib1] Ambrose R.E., Pfeiffer B.E., Foster D.J. (2016). Reverse replay of hippocampal place cells is uniquely modulated by changing reward. Neuron.

[bib2] Behrens T.E.J., Muller T.H., Whittington J.C.R., Mark S., Baram A.B., Stachenfeld K.L., Kurth-Nelson Z. (2018). What Is a Cognitive Map? Organizing Knowledge for Flexible Behavior. Neuron.

[bib3] Bengio Y., Mesnil G., Dauphin Y., Rifai S. (2013). Better mixing via deep representations. PMLR.

[bib4] Bernardi S., Benna M.K., Rigotti M., Munuera J., Fusi S., Salzman D. (2018). The geometry of abstraction in hippocampus and prefrontal cortex. bioRxiv.

[bib5] Botvinick M., Toussaint M. (2012). Planning as inference. Trends Cogn. Sci..

[bib6] Buzsáki G. (2015). Hippocampal sharp wave-ripple: A cognitive biomarker for episodic memory and planning. Hippocampus.

[bib7] Buzsáki G., Moser E.I. (2013). Memory, navigation and theta rhythm in the hippocampal-entorhinal system. Nat. Neurosci..

[bib8] Constantinescu A.O., O’Reilly J.X., Behrens T.E.J. (2016). Organizing conceptual knowledge in humans with a gridlike code. Science.

[bib9] Dalal S., Jerbi K., Bertrand O., Adam C., Ducorps A., Schwartz D., Martinerie J., Lachaux J.-P. (2013). Simultaneous MEG-intracranial EEG: new insights into the ability of MEG to capture oscillatory modulations in the neocortex and the hippocampus. Epilepsy Behav..

[bib10] Davidson T.J., Kloosterman F., Wilson M.A. (2009). Hippocampal replay of extended experience. Neuron.

[bib11] Dehaene S., Meyniel F., Wacongne C., Wang L., Pallier C. (2015). The neural representation of sequences: from transition probabilities to algebraic patterns and linguistic trees. Neuron.

[bib12] Diba K., Buzsáki G. (2007). Forward and reverse hippocampal place-cell sequences during ripples. Nat. Neurosci..

[bib13] Dragoi G., Tonegawa S. (2011). Preplay of future place cell sequences by hippocampal cellular assemblies. Nature.

[bib14] Dragoi G., Tonegawa S. (2013). Distinct preplay of multiple novel spatial experiences in the rat. Proc. Natl. Acad. Sci. USA.

[bib15] Dragoi G., Tonegawa S. (2013). Selection of preconfigured cell assemblies for representation of novel spatial experiences. Philos. Trans. R. Soc. Lond. B Biol. Sci..

[bib16] Eichenbaum H. (2017). Prefrontal-hippocampal interactions in episodic memory. Nat. Rev. Neurosci..

[bib17] Foster D.J. (2017). Replay comes of age. Annu. Rev. Neurosci..

[bib18] Foster D.J., Wilson M.A. (2006). Reverse replay of behavioural sequences in hippocampal place cells during the awake state. Nature.

[bib19] Friston K., Buzsáki G. (2016). The functional anatomy of time: what and when in the brain. Trends Cogn. Sci..

[bib20] Fyhn M., Hafting T., Treves A., Moser M.-B., Moser E.I. (2007). Hippocampal remapping and grid realignment in entorhinal cortex. Nature.

[bib21] Girardeau G., Benchenane K., Wiener S.I., Buzsáki G., Zugaro M.B. (2009). Selective suppression of hippocampal ripples impairs spatial memory. Nat. Neurosci..

[bib22] Grosmark A.D., Buzsáki G. (2016). Diversity in neural firing dynamics supports both rigid and learned hippocampal sequences. Science.

[bib23] Gupta A.S., van der Meer M.A., Touretzky D.S., Redish A.D. (2010). Hippocampal replay is not a simple function of experience. Neuron.

[bib24] Harlow H.F. (1949). The formation of learning sets. Psychol. Rev..

[bib55] Higgins I., Matthey L., Pal A., Burgess C., Glorot X., Botvinick M., Mohamed S., Lerchner A. (2017). beta-VAE: Learning Basic Visual Concepts with a Constrained Variational Framework. ICLR.

[bib25] Higgins I., Pal A., Rusu A.A., Matthey L., Burgess C.P., Pritzel A., Botvinick M., Blundell C., Lerchner A. (2017). Darla: Improving zero-shot transfer in reinforcement learning. arXiv, arXiv:170708475.

[bib26] Hillebrand A., Barnes G.R. (2002). A quantitative assessment of the sensitivity of whole-head MEG to activity in the adult human cortex. Neuroimage.

[bib27] Hsieh L.-T., Gruber M.J., Jenkins L.J., Ranganath C. (2014). Hippocampal activity patterns carry information about objects in temporal context. Neuron.

[bib28] Jadhav S.P., Kemere C., German P.W., Frank L.M. (2012). Awake hippocampal sharp-wave ripples support spatial memory. Science.

[bib29] Jensen O., Gips B., Bergmann T.O., Bonnefond M. (2014). Temporal coding organized by coupled alpha and gamma oscillations prioritize visual processing. Trends Neurosci..

[bib30] Ji D., Wilson M.A. (2007). Coordinated memory replay in the visual cortex and hippocampus during sleep. Nat. Neurosci..

[bib31] Karlsson M.P., Frank L.M. (2009). Awake replay of remote experiences in the hippocampus. Nat. Neurosci..

[bib32] Komorowski R.W., Manns J.R., Eichenbaum H. (2009). Robust conjunctive item-place coding by hippocampal neurons parallels learning what happens here. J. Neurosci..

[bib33] Kornysheva K., Bush D., Meyer S.S., Sadnicka A., Barnes G., Burgess N. (2019). Neural competitive queuing of ordinal structure underlies skilled sequential action. Neuron.

[bib34] Kumaran D., McClelland J.L. (2012). Generalization through the recurrent interaction of episodic memories: a model of the hippocampal system. Psychol. Rev..

[bib35] Kurth-Nelson Z., Barnes G., Sejdinovic D., Dolan R., Dayan P. (2015). Temporal structure in associative retrieval. eLife.

[bib36] Kurth-Nelson Z., Economides M., Dolan R.J., Dayan P. (2016). Fast Sequences of Non-spatial State Representations in Humans. Neuron.

[bib37] Liu K., Sibille J., Dragoi G. (2018). Generative Predictive Codes by Multiplexed Hippocampal Neuronal Tuplets. Neuron.

[bib38] Louie K., Wilson M.A. (2001). Temporally structured replay of awake hippocampal ensemble activity during rapid eye movement sleep. Neuron.

[bib53] Luyckx F., Nili H., Spitzer B., Summerfield C. (2019). Neural structure mapping in human probabilistic reward learning. eLife.

[bib39] Moser E.I., Kropff E., Moser M.-B. (2008). Place cells, grid cells, and the brain’s spatial representation system. Annu. Rev. Neurosci..

[bib40] O’Neill J., Boccara C.N., Stella F., Schoenenberger P., Csicsvari J. (2017). Superficial layers of the medial entorhinal cortex replay independently of the hippocampus. Science.

[bib41] Ólafsdóttir H.F., Barry C., Saleem A.B., Hassabis D., Spiers H.J. (2015). Hippocampal place cells construct reward related sequences through unexplored space. eLife.

[bib42] Ólafsdóttir H.F., Carpenter F., Barry C. (2016). Coordinated grid and place cell replay during rest. Nat. Neurosci..

[bib43] Ólafsdóttir H.F., Carpenter F., Barry C. (2017). Task Demands Predict a Dynamic Switch in the Content of Awake Hippocampal Replay. Neuron.

[bib44] Pezzulo G., van der Meer M.A.A., Lansink C.S., Pennartz C.M.A. (2014). Internally generated sequences in learning and executing goal-directed behavior. Trends Cogn. Sci..

[bib54] Shin H., Lee J.K., Kim J., Kim J. (2017). Continual Learning with Deep Generative Replay. Neural Information Processing Systems.

[bib45] Skaggs W.E., McNaughton B.L. (1996). Replay of neuronal firing sequences in rat hippocampus during sleep following spatial experience. Science.

[bib46] Tenenbaum J.B., Kemp C., Griffiths T.L., Goodman N.D. (2011). How to grow a mind: statistics, structure, and abstraction. Science.

[bib47] Tolman E.C. (1948). Cognitive maps in rats and men. Psychol. Rev..

[bib48] Tsividis, P.A., Pouncy, T., Xu, J.L., Tenenbaum, J.B., and Gershman, S.J. (2017). Human learning in Atari. AAAI Spring Symposium Series.

[bib49] Van Veen B.D., van Drongelen W., Yuchtman M., Suzuki A. (1997). Localization of brain electrical activity via linearly constrained minimum variance spatial filtering. IEEE Trans. Biomed. Eng..

[bib50] Wang J.X., Kurth-Nelson Z., Kumaran D., Tirumala D., Soyer H., Leibo J.Z., Hassabis D., Botvinick M. (2018). Prefrontal cortex as a meta-reinforcement learning system. Nat. Neurosci..

[bib56] Whittington J., Muller T., Mark S., Barry C., Behrens T. (2018). Generalisation of structural knowledge in the hippocampal-entorhinal system. Neural Information Processing Systems.

[bib51] Wimmer G.E., Shohamy D. (2012). Preference by association: how memory mechanisms in the hippocampus bias decisions. Science.

[bib52] Yoon K., Buice M.A., Barry C., Hayman R., Burgess N., Fiete I.R. (2013). Specific evidence of low-dimensional continuous attractor dynamics in grid cells. Nat. Neurosci..

